# Synthesis, characterization, crystal structure and Hirshfeld surface analysis of isobutyl 4-[4-(di­fluoro­meth­oxy)phen­yl]-2,6,6-trimethyl-5-oxo-1,4,5,6,7,8-hexa­hydro­quinoline-3-carboxyl­ate

**DOI:** 10.1107/S2056989023009623

**Published:** 2023-11-10

**Authors:** Sema Öztürk Yıldırım, Mehmet Akkurt, Gökalp Çetin, Rahime Şimşek, Ray J. Butcher, Ajaya Bhattarai

**Affiliations:** a Department of Physics, Faculty of Science, Eskisehir Technical University, Yunus Emre Campus 26470 Eskisehir, Türkiye; b Department of Physics, Faculty of Science, Erciyes University, 38039 Kayseri, Türkiye; c Department of Physics, Faculty of Sciences, Erciyes University, 38039 Kayseri, Türkiye; d Department of Pharmaceutical Chemistry, Faculty of Pharmacy, Erzincan Binali Yıldırım University, 24100 Erzincan, Türkiye; e Department of Pharmaceutical Chemistry, Faculty of Pharmacy, Hacettepe University, 06100 Sıhhiye-Ankara, Türkiye; f Department of Chemistry, Howard University, Washington DC 20059, USA; g Department of Chemistry, M.M.A.M.C (Tribhuvan University), Biratnagar, Nepal; University of Missouri-Columbia, USA

**Keywords:** crystal structure, 1,4-di­hydro­pyridine ring, cyclo­hexene ring, quinoline ring system, van der Waals inter­actions, Hirshfeld surface analysis

## Abstract

In the title compound, C_24_H_29_F_2_NO_4_, which crystallizes in the ortho­rhom­bic *Pca*2_1_ space group with *Z* = 4, the 1,4-di­hydro­pyridine ring adopts a distorted boat conformation, while the cyclo­hexene ring is in a distorted half-chair conformation. In the crystal, the mol­ecules are linked by N—H⋯O and C—H⋯O inter­actions, forming supra­molecular chains parallel to the *a* axis. These chains pack with C—H⋯π inter­actions between them, forming layers parallel to the (010) plane. The cohesion of the crystal structure is ensured by van der Waals inter­actions between these layers. Hirshfeld surface analysis shows the major contributions to the crystal packing are from H⋯H (56.9%), F⋯H/H⋯F (15.7%), O⋯H/H⋯O (13.7%) and C⋯H/H⋯C (9.5%) contacts.

## Abstract 2

In the crystal, the mol­ecules are linked by N—H⋯O and C—H⋯O inter­actions, forming supra­molecular chains parallel to the *a* axis. These chains pack with C—H⋯π inter­actions between them, forming layers parallel to the (010) plane.

## 1. Chemical context

Hexa­hydro­quinoline (HHQ) ring systems occupy a prominent place in medicinal chemistry, attracting the attention of researchers for their versatile structural attributes and pharmacological potential. These ring systems, characterized by a unique combination of pyridine and cyclo­hexane rings, have shown remarkable bioactivity across a spectrum of therapeutic areas. Their capacity to inter­act with specific biological targets has led to the development of HHQ-based compounds with diverse medicinal properties, including anti­microbial, anti-inflammatory, and anti­cancer activities (Ranjbar *et al.*, 2019 ▸). Recent studies have shown that these compounds are effective in cancer-related inflammatory pathways such as TGF-β (Längle *et al.*, 2019 ▸). Additionally, they have been demonstrated to have inhibitory effects on receptors involved in cancer development, such as EGFR, or to reverse multi-drug resistance (Abo Al-Hamd *et al.*, 2023 ▸; Shahraki *et al.*, 2020 ▸).

The choice to synthesize HHQs is also fueled by the accessibility of various synthetic routes and the opportunity to fine-tune their chemical structure to optimize drug-like properties. Multi-component reactions and cyclization strategies provide versatile platforms for their synthesis, allowing for systematic modifications to explore structure–activity relationships (SAR; Batista *et al.*, 2016 ▸). As a result, the strategic pursuit of hexa­hydro­quinoline synthesis continues to be a compelling avenue in medicinal chemistry, promising innovative solutions to pressing medical challenges and drug discovery endeavors. 
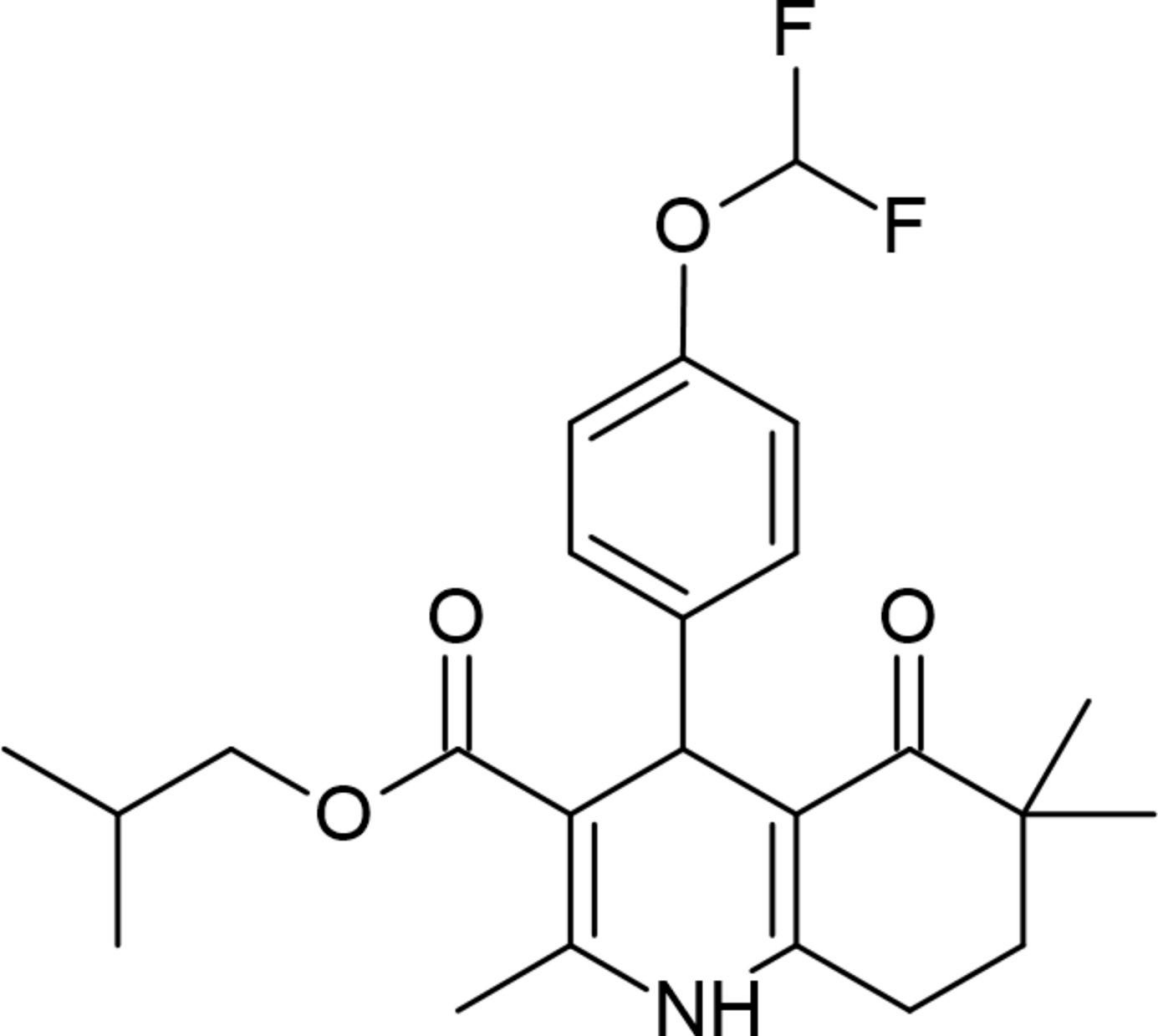


In this study, isobutyl 4-(4-di­fluoro­meth­oxy­phen­yl)-2,6,6-trimethyl-5-oxo-1,4,5,6,7,8-hexa­hydro­quinoline-3-carb­oxyl­ate was synthesized and its mol­ecular structure was confirmed by IR, ^1^H NMR, ^13^C NMR, HRMS and X-ray crystallography. The inter­molecular inter­actions observed in the crystal packing were investigated by Hirshfeld surface analysis.

## 2. Structural commentary

**Figure 1:**
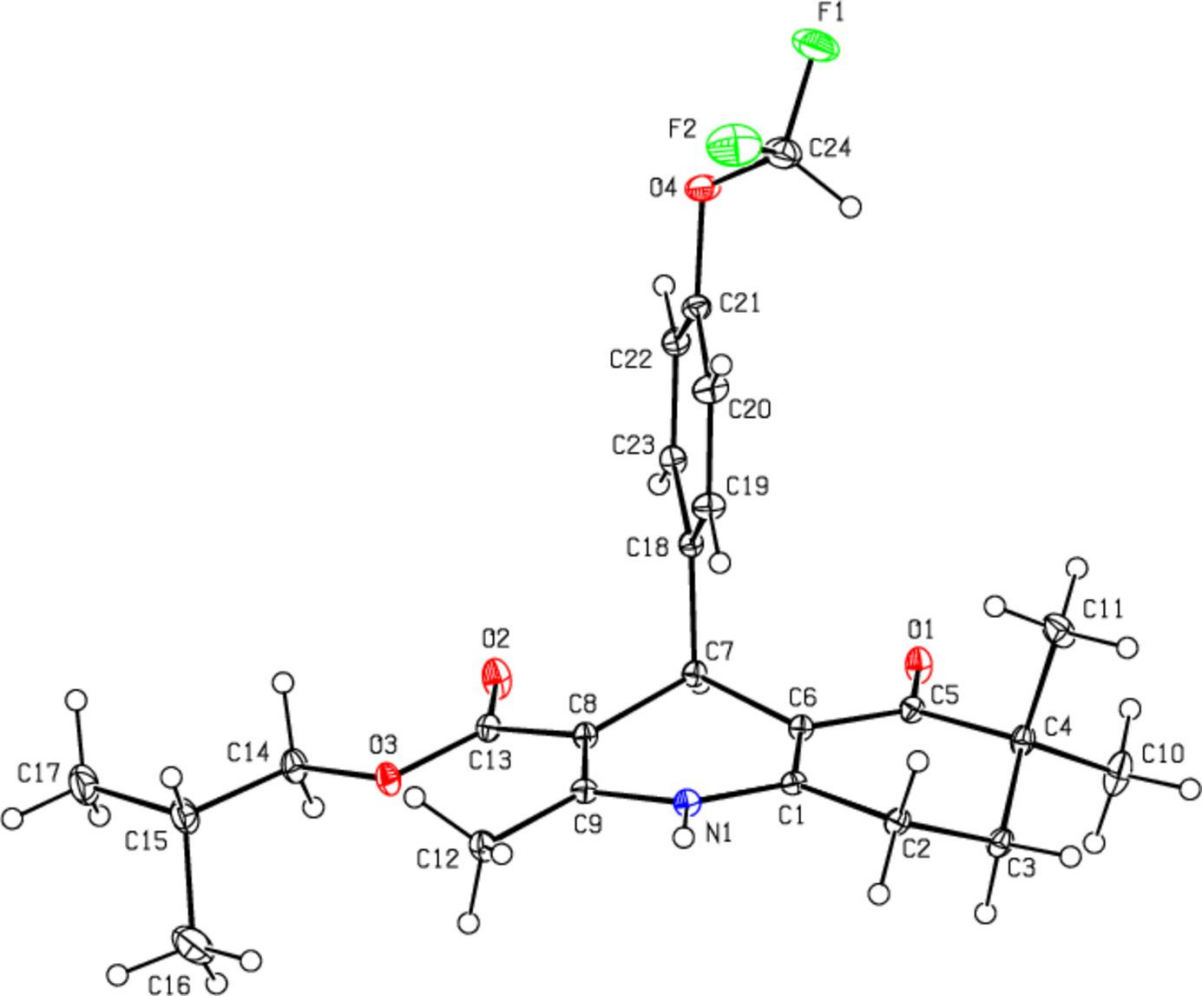
View of the title mol­ecule. Displacement ellipsoids are drawn at the 30% probability level.

The 1,4-di­hydro­pyridine ring (N1/C1/C6–C9) of the title compound (Fig. 1 ▸), which crystallizes in the ortho­rhom­bic *P*ca2_1_ space group with *Z* = 4, adopts a distorted boat conformation [puckering parameters (Cremer & Pople, 1975 ▸) are *Q*_T_ = 0.2779 (16) Å, θ = 73.7 (3)° and φ = 179.1 (3)°], while the cyclo­hexene ring (C1–C6) has a distorted half-chair conformation [puckering parameters are *Q*_T_ = 0.4464 (18) Å, θ = 48.9 (2)° and φ = 126.3 (3)°]. The 4-(4-di­fluoro­meth­oxy­phenyl) ring (C18–C23) makes a dihedral angle of 88.73 (6)° with the mean plane of the quinoline ring system [N1/C1–C9; maximum deviation = 0.415 (2) Å for C3]. The geometrical parameters of the title compound are in agreement with those reported for similar compounds in the *Database survey* section.

## 3. Supra­molecular features and Hirshfeld surface analysis

**Figure 2:**
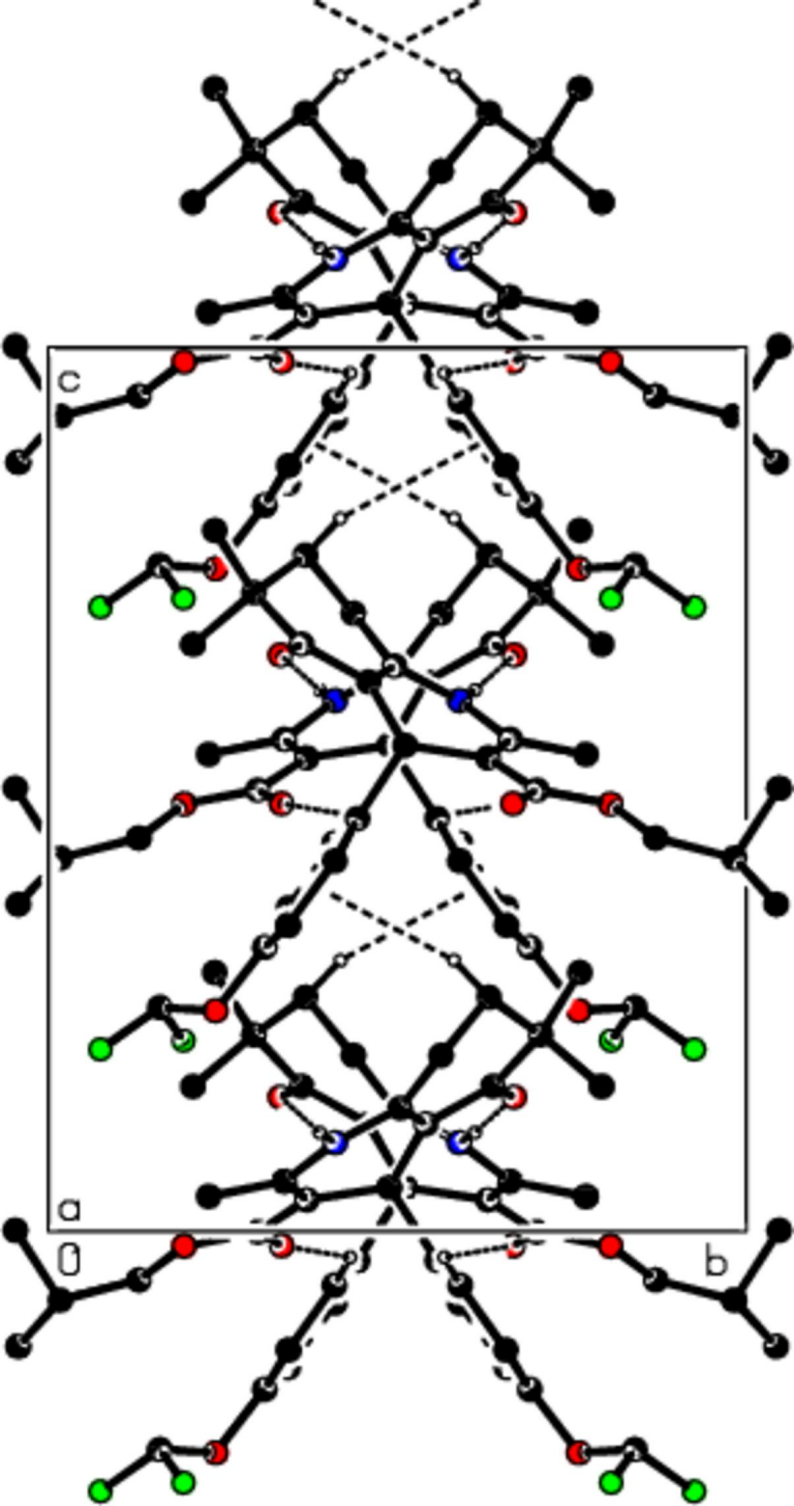
A view of the mol­ecular packing of the title compound along the *a*-axis with the N—H⋯O, C—H⋯O hydrogen bonds and C—H⋯π inter­actions shown as dashed lines.

**Figure 4:**
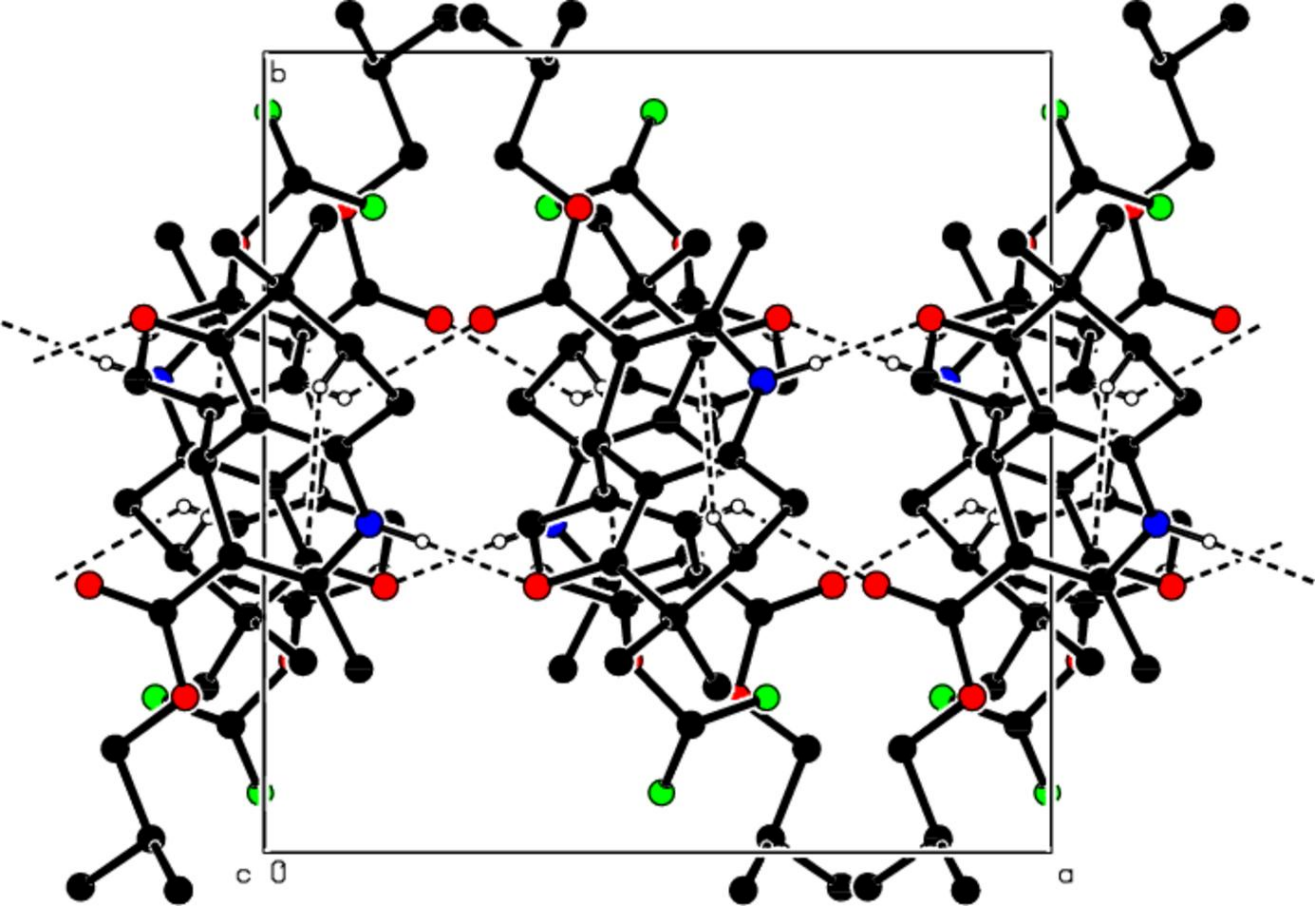
View of the mol­ecular packing along the *c*-axis. Hydrogen bonds are shown as dashed lines.

In the crystal, the mol­ecules are linked by N—H⋯O and C—H⋯O inter­actions, forming supra­molecular chains parallel to the *a*-axis direction (see Table 1 ▸; Figs. 2 ▸ and 3 ▸). These chains pack with C—H⋯π inter­actions between them, forming layers parallel to the (010) plane (Fig. 4 ▸). The cohesion of the crystal structure is ensured by van der Waals inter­actions between these layers.

**Figure 5:**
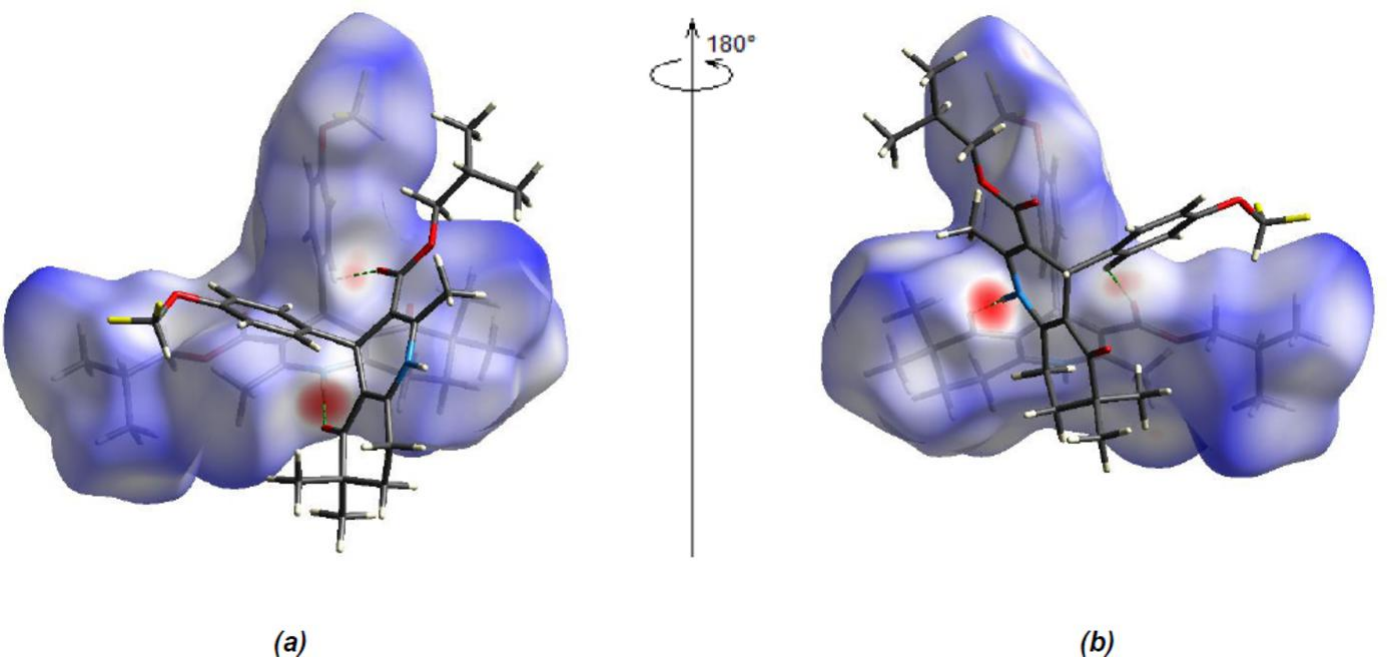
(*a*) Front and (*b*) back views of the three-dimensional Hirshfeld surface for the title compound.

The Hirshfeld surfaces and their corresponding two-dimensional fingerprint plots were calculated using the software package *Crystal Explorer 17.5* (Spackman *et al.*, 2021 ▸). The *d*_norm_ surfaces are mapped over a fixed color scale from −0.5961 (red) to 1.9017 (blue) a.u. Red spots on the surface correspond to O⋯H/H⋯O inter­actions (Tables 1 ▸ and 2 ▸; Fig. 5 ▸ *a*,*b*).

**Figure 6:**
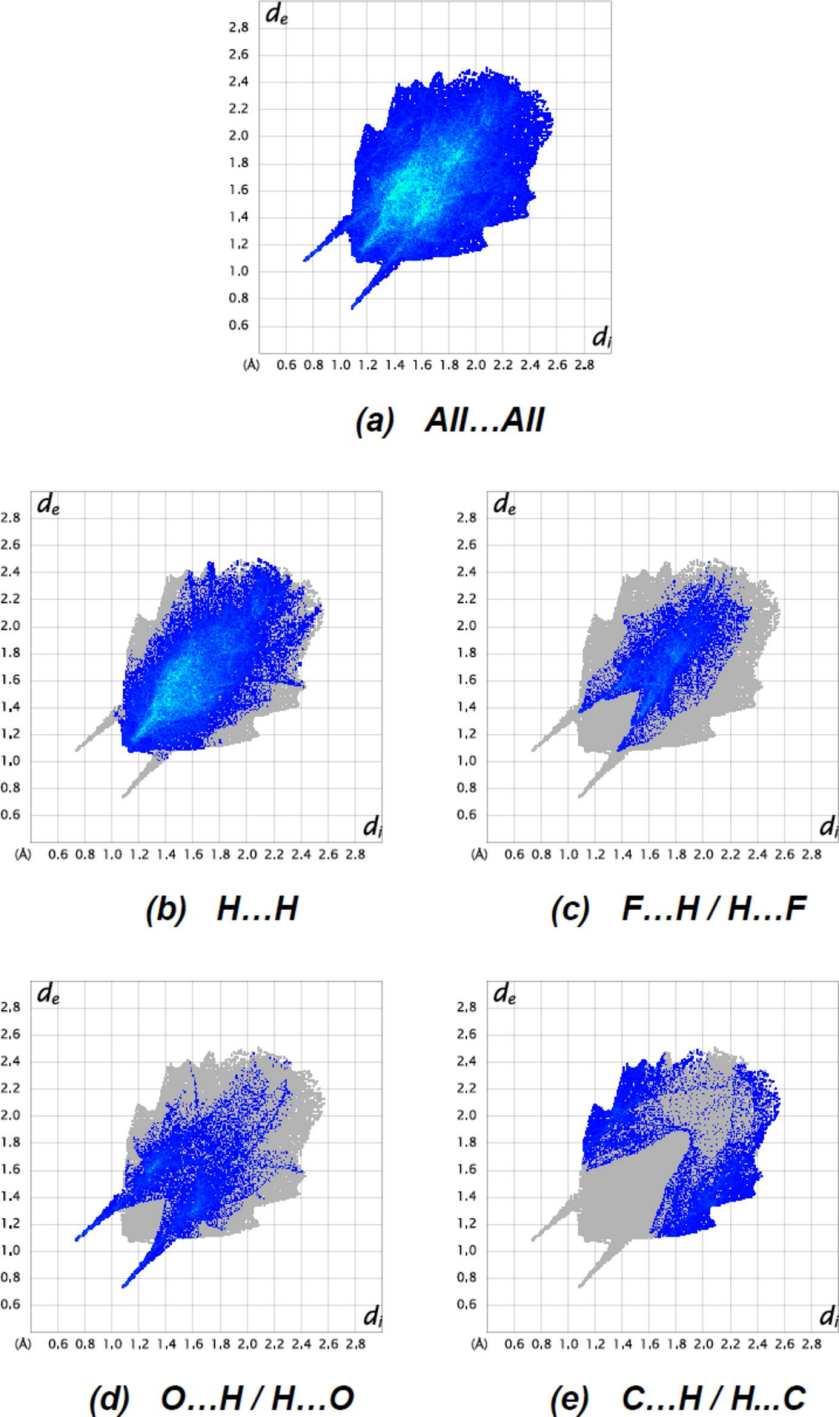
The two-dimensional fingerprint plots for the title compound showing (*a*) all inter­actions, and delineated into (*b*) H⋯H, (*c*) F⋯H/H⋯F, (*d*) O⋯H/H⋯O and (*e*) C⋯H/H⋯C inter­actions. The *d*_i_ and *d*_e_ values are the closest inter­nal and external distances (in Å) from given points on the Hirshfeld surface.

**Figure 7:**
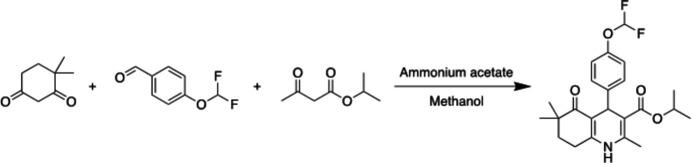
Synthetic scheme.

In Fig. 6 ▸, fingerprint plots of the most important non-covalent inter­actions for the title compound are shown. The major contributions to the crystal packing are from H⋯H (56.9%), F⋯H/H⋯F (15.7%), O⋯H/H⋯O (13.7%) and C⋯H/H⋯C (9.5%) contacts. O⋯C/C⋯O (1.1%), F⋯C/C⋯F (1.0%), C⋯C (0.7%), F⋯O/O⋯F (0.6%), O⋯N/N⋯O (0.5%) and N⋯H/H⋯N (0.2%) contacts, which contribute less than 1.1%, are not shown in Fig.7.

## 4. Database survey

A search of the Cambridge Structural Database (CSD, Version 5.42, update of September 2021; Groom *et al.*, 2016 ▸) for similar structures with the 1,4,5,6,7,8-hexa­hydro­quinoline group showed that the nine most closely related to the title compound are LIMYUF (Pehlivanlar *et al.*, 2023 ▸), WEZJUK (Yıldırım *et al.*, 2023 ▸), ECUCUE (Yıldırım *et al.*, 2022 ▸), LOQCAX (Steiger *et al.*, 2014 ▸), NEQMON (Öztürk Yildirim, *et al.*, 2013 ▸), PECPUK (Gündüz *et al.*, 2012 ▸), IMEJOA (Linden *et al.*, 2011 ▸), PUGCIE (Mookiah *et al.*, 2009 ▸), UCOLOO (Linden *et al.*, 2006 ▸) and DAYJET (Linden *et al.*, 2005 ▸). In all of these compounds, mol­ecules are linked by N—H⋯O hydrogen bonds. Furthermore, C—H⋯F hydrogen bonds in LIMYUF, C—H⋯O hydrogen bonds in WEZJUK, ECUCUE, NEQMON, IMEJOA and PUGCIE and C—H⋯π inter­actions in LIMYUF, WEZJUK and ECUCUE were also observed.

## 5. Synthesis and crystallization

The synthesis of the compound was carried out by refluxing 1 mmol of 4-(4-di­fluoro­meth­oxy)benzaldehyde, isobutyl aceto­acetate, 4,4-methyl-1,3-cyclo­hexa­ndione and 5 mmol of ammonium acetate in methanol. The reaction process was monitored by thin-layer chromatography [ethyl acetate-*n*-hexane (1:1)], and after the reaction was complete, the mixture was allowed to stand at room temperature for a while and then poured into an ice–water mixture (Fig. 7 ▸). The resulting precipitates were purified again by crystallization with methanol (Yıldırım *et al.*, 2023 ▸).

***Isobutyl 4-(4-di­fluoro­meth­oxy­phen­yl)-2,6,6-tri­methyl-5-oxo-1,4,5,6,7,8-hexa­hydro­quinoline-3-carbox­y­l­ate***

Light-yellow solid, m.p: 489–491 K, yield: 85%. IR (cm^−1^) 3291 (N—H), 1674 (C=O, ester), 1597 (C=O, ketone). ^1^H NMR (400 MHz, DMSO-*d*_6_): δ 0.69 [3H, *d*, *J* = 9 Hz, –CH(**CH_3_**)_a_], 0.77 [3H, *d*, *J* = 9 Hz, –CH(**CH_3_**)_b_], 0.81 (3H, *s*, 6-CH_3_), 0.97 (3H, *s*, 6-CH_3_), 1.52–1.65 (2H, *m*, quinoline H_7_), 1.72–1.81 (H, *m*, –CH–), 2.26 (3H, *s*, 2-CH_3_), 2.46–2.50 (2H, *m*, quinoline H_8_), 3.63–3.72 (2H, *m*, –**CH_2_**)–, 4.94 (H, *s*, quinoline H_4_), 6.96 (2H, *dd*, *J* = 9.2, 6.8 Hz, Ar-H_3,5_), 7.16 (2H, *dd*, *J* = 9.2, 6.8 Hz, Ar-H_4,6_), 7.26 (H, *s*, O**CH**F_2_), 9.02 (H, *s*, NH). ^13^C NMR (100 MHz, DMSO-*d*_6_): 18.6 (2-CH_3_), 22.3 [–CH(**CH_3_**)_*a*_], 22.9 [–CH(**CH_3_**)_*b*_], 23.4 (C-8), 24.2 (6-CH_3_), 24.8 (6-CH_3_), 33.5 (C-7), 34.1 (C-4), 35 (–CH–), 39.5 (C-6), 68.2 (–CH_2_–), 103.8 (C-3), 108.4 (C-4a), 114.2, 116.7, 125.4, 128.2, 135.5, 157.4 (phenyl carbons), 147.1(C-2), 150.6 (C-8a), 166.9 (–COO–), 168.3 (OCHF_2_) 199.6 (C-5). HRMS (ESI/Q-TOF) *m*/*z*: [*M* + H]^+^ calculated for C_23_H_25_F_4_NO_3_: 420.1942; found: 420.2150.

## 6. Refinement

Crystal data, data collection and structure refinement details are summarized in Table 3 ▸. The N-bound H atom was located in a difference-Fourier map and refined freely [N1—H1*N* = 0.87 (2) Å]. All C-bound H atoms were positioned geometrically [C—H = 0.95–1.00 Å] and refined using a riding model with *U*_iso_(H) = 1.2 or 1.5*U*_eq_(C).

## Supplementary Material

Crystal structure: contains datablock(s) I. DOI: 10.1107/S2056989023009623/ev2001sup1.cif

Structure factors: contains datablock(s) I. DOI: 10.1107/S2056989023009623/ev2001Isup2.hkl

Supporting information file. DOI: 10.1107/S2056989023009623/ev2001Isup3.cml

CCDC reference: 2305562

Additional supporting information:  crystallographic information; 3D view; checkCIF report

## Figures and Tables

**Figure 3:**
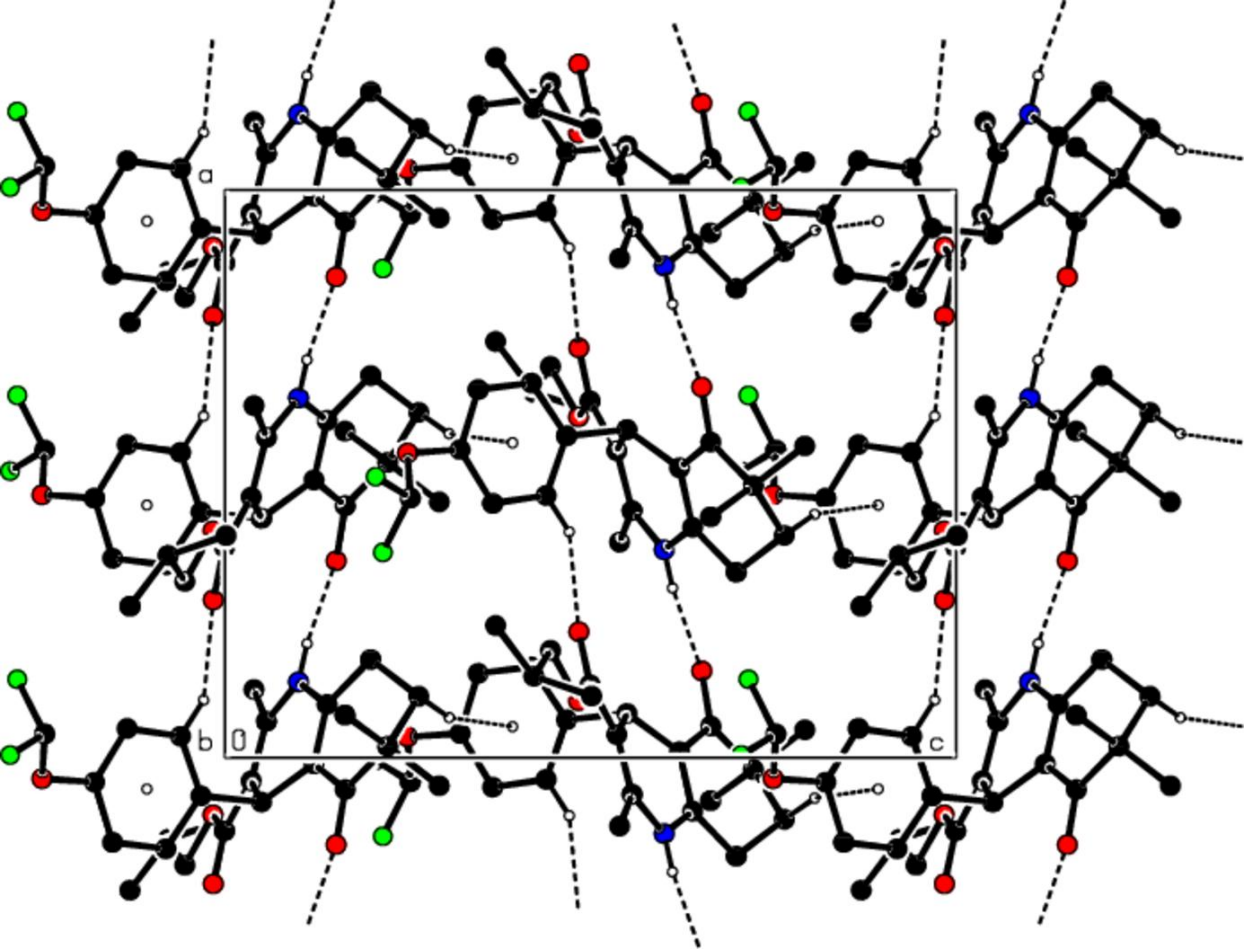
View of the mol­ecular packing along the *b*-axis. Hydrogen bonds are shown as dashed lines.

**Table 1:** Hydrogen-bond geometry (Å, °) *Cg*3 is the centroid the benzene ring of the 4-(4-di­fluoro­meth­oxy­phenyl group of the title compound.

*D*—H⋯*A*	*D*—H	H⋯*A*	*D*⋯*A*	*D*—H⋯*A*
N1—H1*N*⋯O1^i^	0.87 (2)	1.98 (2)	2.8426 (17)	173 (2)
C19—H19*A*⋯O2^i^	0.95	2.43	3.100 (2)	127
C3—H3*A*⋯*Cg*3^ii^	0.99	2.84	3.7345 (18)	151

Symmetry codes: (i) 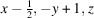 ; (ii) 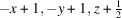 .

**Table 2:** Summary of short inter­atomic contacts (Å) in the title compound

H15*A*⋯H10*B*	2.37	1 − *x*, 1 − *y*, − 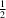 + *z*
F2⋯H11*B*	2.83	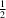 − *x*, *y*, − 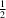 + *z*
O1⋯H1*N*	1.98	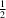 + *x*, 1 − *y*, *z*
H10*A*⋯H22*A*	2.47	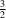 − *x*, *y*, 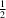 + *z*
H17*B*⋯H22*A*	2.58	*x*, −1 + *y*, *z*
H12*A*⋯H17*C*	2.54	− 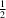 + *x*, −*y*, *z*

**Table 3:** Experimental details

Crystal data	
Chemical formula	C_24_H_29_F_2_NO_4_
*M*_r_	433.48
Crystal system, space group	Orthorhombic, *P* *c* *a*2_1_
Temperature (K)	100
*a*, *b*, *c* (Å)	11.9879 (7), 12.1807 (7), 15.4518 (9)
*V* (Å^3^)	2256.3 (2)
*Z*	4
Radiation type	Mo *K*α
μ (mm^−1^)	0.10
Crystal size (mm)	0.27 × 0.24 × 0.16

Data collection	
Diffractometer	Bruker Quest D8 with Photon 2 detector
Absorption correction	Multi-scan (*SADABS*; Krause *et al.*, 2015 ▸)
*T*_min_, *T*_max_	0.718, 0.744
No. of measured, independent and observed [*I* > 2σ(*I*)] reflections	103269, 9491, 7264
*R*_int_	0.080
(sin θ/λ)_max_ (Å^−1^)	0.826

Refinement	
*R*[*F* ^2^ > 2σ(*F* ^2^)], *wR*(*F* ^2^), *S*	0.045, 0.107, 1.03
No. of reflections	9491
No. of parameters	289
No. of restraints	1
H-atom treatment	H atoms treated by a mixture of independent and constrained refinement
Δρ_max_, Δρ_min_ (e Å^−3^)	0.32, −0.29
Absolute structure	Flack *x* determined using 2721 quotients [(*I* ^+^)−(*I* ^−^)]/[(*I* ^+^)+(*I* ^−^)] (Parsons *et al.*, 2013 ▸)
Absolute structure parameter	0.0 (2)

Computer programs: *APEX2* and *SAINT* (Bruker, 2018 ▸), *SHELXT* (Sheldrick, 2015*a* ▸), *SHELXL* (Sheldrick, 2015*b* ▸), *ORTEP-3 for Windows* (Farrugia, 2012 ▸) and *PLATON* (Spek, 2020 ▸).

## supplementary crystallographic information

### Crystal data

**Table:**

C_24_H_29_F_2_NO_4_	*D*_x_ = 1.276 Mg m^−^^3^
*M**_r_* = 433.48	Mo *K*α radiation, λ = 0.71073 Å
Orthorhombic, *P**c**a*2_1_	Cell parameters from 9859 reflections
*a* = 11.9879 (7) Å	θ = 2.4–32.7°
*b* = 12.1807 (7) Å	µ = 0.10 mm^−^^1^
*c* = 15.4518 (9) Å	*T* = 100 K
*V* = 2256.3 (2) Å^3^	Chunk, light yellow
*Z* = 4	0.27 × 0.24 × 0.16 mm
*F*(000) = 920	

### Data collection

**Table:**

Bruker Quest D8 with Photon 2 detector diffractometer	7264 reflections with *I* > 2σ(*I*)
φ and ω scans	*R*_int_ = 0.080
Absorption correction: multi-scan (SADABS; Krause *et al.*, 2015)	θ_max_ = 36.0°, θ_min_ = 2.4°
*T*_min_ = 0.718, *T*_max_ = 0.744	*h* = −18→19
103269 measured reflections	*k* = −20→19
9491 independent reflections	*l* = −25→21

### Refinement

**Table:**

Refinement on *F*^2^	Hydrogen site location: mixed
Least-squares matrix: full	H atoms treated by a mixture of independent and constrained refinement
*R*[*F*^2^ > 2σ(*F*^2^)] = 0.045	*w* = 1/[σ^2^(*F*_o_^2^) + (0.0508*P*)^2^ + 0.3823*P*] where *P* = (*F*_o_^2^ + 2*F*_c_^2^)/3
*wR*(*F*^2^) = 0.107	(Δ/σ)_max_ < 0.001
*S* = 1.03	Δρ_max_ = 0.32 e Å^−^^3^
9491 reflections	Δρ_min_ = −0.29 e Å^−^^3^
289 parameters	Absolute structure: Flack *x* determined using 2721 quotients [(*I*^+^)-(*I*^-^)]/[(*I*^+^)+(*I*^-^)] (Parsons *et al.*, 2013)
1 restraint	Absolute structure parameter: 0.0 (2)

### Special details

**Table:**

Geometry. All esds (except the esd in the dihedral angle between two l.s. planes) are estimated using the full covariance matrix. The cell esds are taken into account individually in the estimation of esds in distances, angles and torsion angles; correlations between esds in cell parameters are only used when they are defined by crystal symmetry. An approximate (isotropic) treatment of cell esds is used for estimating esds involving l.s. planes.

### Fractional atomic coordinates and isotropic or equivalent isotropic displacement parameters (Å^2^)

**Table:**

	*x*	*y*	*z*	*U*_iso_*/*U*_eq_	
F1	0.49509 (14)	0.92511 (11)	0.20621 (10)	0.0455 (4)	
F2	0.36176 (12)	0.80630 (13)	0.21602 (10)	0.0467 (4)	
O1	0.65307 (10)	0.66738 (10)	0.65205 (8)	0.0198 (2)	
O2	0.72200 (10)	0.33422 (10)	0.48402 (9)	0.0239 (3)	
O3	0.60039 (10)	0.19451 (9)	0.48360 (8)	0.0184 (2)	
O4	0.53670 (11)	0.76006 (11)	0.24983 (8)	0.0236 (3)	
N1	0.36644 (10)	0.41080 (11)	0.60065 (9)	0.0143 (2)	
H1N	0.299 (2)	0.3886 (19)	0.6122 (14)	0.019 (5)*	
C1	0.40589 (12)	0.50413 (12)	0.63969 (10)	0.0128 (3)	
C2	0.32686 (12)	0.56285 (13)	0.69932 (10)	0.0149 (3)	
H2A	0.279873	0.508580	0.730076	0.018*	
H2B	0.277189	0.611193	0.665143	0.018*	
C3	0.39163 (14)	0.63141 (13)	0.76504 (11)	0.0171 (3)	
H3A	0.429018	0.581636	0.806558	0.021*	
H3B	0.338594	0.677625	0.797973	0.021*	
C4	0.47947 (13)	0.70546 (13)	0.72264 (11)	0.0165 (3)	
C5	0.55598 (12)	0.63711 (12)	0.66451 (10)	0.0142 (3)	
C6	0.51077 (12)	0.54097 (12)	0.62178 (10)	0.0134 (3)	
C7	0.57801 (12)	0.48870 (12)	0.54998 (10)	0.0129 (2)	
H7A	0.658250	0.488219	0.567679	0.015*	
C8	0.54078 (12)	0.37018 (12)	0.53589 (10)	0.0135 (3)	
C9	0.43551 (13)	0.33826 (12)	0.55678 (10)	0.0142 (3)	
C10	0.54927 (15)	0.76147 (18)	0.79303 (13)	0.0285 (4)	
H10A	0.584192	0.705497	0.829657	0.043*	
H10B	0.500970	0.808215	0.828535	0.043*	
H10C	0.607286	0.806523	0.765913	0.043*	
C11	0.42455 (16)	0.79274 (14)	0.66419 (14)	0.0246 (4)	
H11A	0.482159	0.841376	0.640633	0.037*	
H11B	0.371361	0.835939	0.698245	0.037*	
H11C	0.385240	0.756348	0.616550	0.037*	
C12	0.37972 (13)	0.22972 (13)	0.53973 (11)	0.0186 (3)	
H12A	0.400712	0.203315	0.482072	0.028*	
H12B	0.298580	0.238862	0.542595	0.028*	
H12C	0.403522	0.176294	0.583404	0.028*	
C13	0.62870 (13)	0.30006 (12)	0.49936 (10)	0.0150 (3)	
C14	0.68925 (15)	0.12977 (14)	0.44523 (12)	0.0208 (3)	
H14A	0.751816	0.122384	0.486642	0.025*	
H14B	0.717478	0.166562	0.392459	0.025*	
C15	0.64362 (16)	0.01733 (14)	0.42246 (14)	0.0260 (4)	
H15A	0.576642	0.027434	0.384717	0.031*	
C16	0.6088 (3)	−0.04712 (19)	0.5015 (2)	0.0529 (8)	
H16A	0.544199	−0.011657	0.528503	0.079*	
H16B	0.670705	−0.049424	0.542902	0.079*	
H16C	0.588934	−0.122088	0.484414	0.079*	
C17	0.7331 (2)	−0.04286 (16)	0.37018 (15)	0.0330 (4)	
H17A	0.747606	−0.002683	0.316403	0.050*	
H17B	0.706990	−0.117084	0.356413	0.050*	
H17C	0.801872	−0.047357	0.404232	0.050*	
C18	0.56699 (12)	0.55695 (12)	0.46724 (10)	0.0137 (3)	
C19	0.46204 (13)	0.59010 (14)	0.43930 (11)	0.0184 (3)	
H19A	0.398046	0.567489	0.470878	0.022*	
C20	0.44827 (14)	0.65559 (15)	0.36621 (12)	0.0208 (3)	
H20A	0.375930	0.676416	0.347231	0.025*	
C21	0.54288 (14)	0.68982 (13)	0.32165 (11)	0.0183 (3)	
C22	0.64869 (13)	0.65619 (13)	0.34636 (11)	0.0175 (3)	
H22A	0.712375	0.678221	0.314155	0.021*	
C23	0.66020 (13)	0.58949 (13)	0.41925 (11)	0.0159 (3)	
H23A	0.732370	0.565862	0.436503	0.019*	
C24	0.45780 (18)	0.84020 (16)	0.25354 (14)	0.0294 (4)	
H24A	0.444326	0.863192	0.314777	0.035*	

### Atomic displacement parameters (Å^2^)

**Table:**

	*U*^11^	*U*^22^	*U*^33^	*U*^12^	*U*^13^	*U*^23^
F1	0.0608 (9)	0.0302 (6)	0.0454 (8)	0.0086 (6)	0.0104 (7)	0.0181 (6)
F2	0.0317 (7)	0.0612 (9)	0.0474 (8)	0.0089 (6)	−0.0074 (6)	0.0154 (7)
O1	0.0120 (5)	0.0199 (5)	0.0276 (6)	−0.0016 (4)	0.0042 (4)	−0.0068 (5)
O2	0.0134 (5)	0.0212 (5)	0.0371 (7)	−0.0005 (4)	0.0070 (5)	−0.0075 (5)
O3	0.0158 (5)	0.0142 (5)	0.0252 (6)	0.0018 (4)	0.0030 (4)	−0.0036 (4)
O4	0.0262 (6)	0.0255 (6)	0.0191 (6)	0.0043 (5)	0.0044 (5)	0.0068 (5)
N1	0.0093 (5)	0.0162 (6)	0.0173 (6)	−0.0009 (4)	0.0014 (5)	−0.0004 (5)
C1	0.0110 (6)	0.0149 (6)	0.0124 (6)	0.0015 (5)	0.0006 (5)	0.0011 (5)
C2	0.0116 (6)	0.0179 (6)	0.0153 (7)	0.0009 (5)	0.0033 (5)	−0.0012 (5)
C3	0.0151 (7)	0.0203 (7)	0.0158 (7)	−0.0002 (6)	0.0034 (5)	−0.0026 (6)
C4	0.0129 (6)	0.0187 (7)	0.0177 (7)	−0.0003 (5)	0.0031 (5)	−0.0051 (5)
C5	0.0122 (7)	0.0156 (6)	0.0148 (6)	0.0015 (5)	0.0004 (5)	−0.0007 (5)
C6	0.0113 (6)	0.0143 (6)	0.0145 (6)	0.0004 (5)	0.0005 (5)	−0.0005 (5)
C7	0.0095 (6)	0.0147 (6)	0.0145 (6)	0.0001 (5)	0.0017 (5)	−0.0013 (5)
C8	0.0110 (6)	0.0141 (6)	0.0154 (6)	0.0006 (5)	0.0009 (5)	−0.0011 (5)
C9	0.0130 (6)	0.0151 (6)	0.0146 (7)	0.0004 (5)	−0.0001 (5)	0.0012 (5)
C10	0.0201 (8)	0.0368 (10)	0.0285 (9)	−0.0063 (7)	0.0049 (7)	−0.0175 (8)
C11	0.0202 (8)	0.0184 (7)	0.0353 (10)	0.0029 (6)	0.0080 (7)	0.0023 (7)
C12	0.0159 (7)	0.0172 (7)	0.0229 (8)	−0.0034 (5)	0.0021 (6)	−0.0016 (6)
C13	0.0141 (6)	0.0153 (6)	0.0156 (7)	0.0010 (5)	−0.0002 (5)	−0.0020 (5)
C14	0.0185 (7)	0.0177 (7)	0.0261 (8)	0.0051 (6)	0.0032 (6)	−0.0039 (6)
C15	0.0286 (9)	0.0163 (7)	0.0331 (10)	0.0030 (6)	0.0065 (7)	−0.0029 (7)
C16	0.0751 (19)	0.0233 (10)	0.0603 (17)	0.0074 (11)	0.0366 (15)	0.0097 (10)
C17	0.0388 (11)	0.0202 (8)	0.0401 (11)	0.0047 (7)	0.0097 (9)	−0.0055 (8)
C18	0.0115 (6)	0.0139 (6)	0.0157 (7)	−0.0003 (5)	0.0021 (5)	−0.0013 (5)
C19	0.0124 (6)	0.0234 (7)	0.0195 (7)	0.0007 (6)	0.0024 (5)	0.0035 (6)
C20	0.0144 (7)	0.0279 (8)	0.0201 (8)	0.0024 (6)	0.0000 (6)	0.0044 (7)
C21	0.0202 (7)	0.0183 (7)	0.0164 (7)	0.0010 (6)	0.0036 (6)	0.0019 (6)
C22	0.0160 (7)	0.0176 (7)	0.0190 (7)	−0.0020 (5)	0.0049 (6)	−0.0007 (6)
C23	0.0118 (6)	0.0160 (6)	0.0200 (7)	−0.0013 (5)	0.0023 (5)	−0.0015 (5)
C24	0.0336 (10)	0.0265 (9)	0.0280 (10)	0.0059 (7)	0.0031 (8)	0.0076 (7)

### Geometric parameters (Å, º)

**Table:**

F1—C24	1.343 (2)	C10—H10B	0.9800
F2—C24	1.354 (3)	C10—H10C	0.9800
O1—C5	1.2361 (19)	C11—H11A	0.9800
O2—C13	1.2166 (19)	C11—H11B	0.9800
O3—C13	1.3518 (18)	C11—H11C	0.9800
O3—C14	1.4520 (19)	C12—H12A	0.9800
O4—C24	1.360 (2)	C12—H12B	0.9800
O4—C21	1.403 (2)	C12—H12C	0.9800
N1—C1	1.371 (2)	C14—C15	1.516 (2)
N1—C9	1.388 (2)	C14—H14A	0.9900
N1—H1N	0.87 (2)	C14—H14B	0.9900
C1—C6	1.363 (2)	C15—C16	1.511 (3)
C1—C2	1.503 (2)	C15—C17	1.530 (3)
C2—C3	1.527 (2)	C15—H15A	1.0000
C2—H2A	0.9900	C16—H16A	0.9800
C2—H2B	0.9900	C16—H16B	0.9800
C3—C4	1.533 (2)	C16—H16C	0.9800
C3—H3A	0.9900	C17—H17A	0.9800
C3—H3B	0.9900	C17—H17B	0.9800
C4—C5	1.530 (2)	C17—H17C	0.9800
C4—C10	1.532 (2)	C18—C19	1.390 (2)
C4—C11	1.543 (2)	C18—C23	1.398 (2)
C5—C6	1.449 (2)	C19—C20	1.392 (2)
C6—C7	1.512 (2)	C19—H19A	0.9500
C7—C8	1.527 (2)	C20—C21	1.391 (2)
C7—C18	1.531 (2)	C20—H20A	0.9500
C7—H7A	1.0000	C21—C22	1.386 (2)
C8—C9	1.359 (2)	C22—C23	1.396 (2)
C8—C13	1.469 (2)	C22—H22A	0.9500
C9—C12	1.505 (2)	C23—H23A	0.9500
C10—H10A	0.9800	C24—H24A	1.0000
			
C13—O3—C14	113.95 (12)	C9—C12—H12B	109.5
C24—O4—C21	116.16 (14)	H12A—C12—H12B	109.5
C1—N1—C9	122.48 (13)	C9—C12—H12C	109.5
C1—N1—H1N	119.2 (15)	H12A—C12—H12C	109.5
C9—N1—H1N	117.1 (15)	H12B—C12—H12C	109.5
C6—C1—N1	120.11 (14)	O2—C13—O3	121.40 (14)
C6—C1—C2	123.32 (14)	O2—C13—C8	122.38 (14)
N1—C1—C2	116.55 (13)	O3—C13—C8	116.23 (13)
C1—C2—C3	110.33 (12)	O3—C14—C15	108.70 (14)
C1—C2—H2A	109.6	O3—C14—H14A	109.9
C3—C2—H2A	109.6	C15—C14—H14A	109.9
C1—C2—H2B	109.6	O3—C14—H14B	109.9
C3—C2—H2B	109.6	C15—C14—H14B	109.9
H2A—C2—H2B	108.1	H14A—C14—H14B	108.3
C2—C3—C4	112.76 (13)	C16—C15—C14	112.43 (19)
C2—C3—H3A	109.0	C16—C15—C17	111.82 (17)
C4—C3—H3A	109.0	C14—C15—C17	107.61 (16)
C2—C3—H3B	109.0	C16—C15—H15A	108.3
C4—C3—H3B	109.0	C14—C15—H15A	108.3
H3A—C3—H3B	107.8	C17—C15—H15A	108.3
C5—C4—C10	109.37 (13)	C15—C16—H16A	109.5
C5—C4—C3	110.02 (13)	C15—C16—H16B	109.5
C10—C4—C3	109.49 (14)	H16A—C16—H16B	109.5
C5—C4—C11	106.68 (13)	C15—C16—H16C	109.5
C10—C4—C11	109.99 (15)	H16A—C16—H16C	109.5
C3—C4—C11	111.25 (13)	H16B—C16—H16C	109.5
O1—C5—C6	121.47 (14)	C15—C17—H17A	109.5
O1—C5—C4	119.58 (14)	C15—C17—H17B	109.5
C6—C5—C4	118.88 (13)	H17A—C17—H17B	109.5
C1—C6—C5	121.21 (14)	C15—C17—H17C	109.5
C1—C6—C7	120.13 (14)	H17A—C17—H17C	109.5
C5—C6—C7	118.37 (13)	H17B—C17—H17C	109.5
C6—C7—C8	110.30 (12)	C19—C18—C23	118.43 (15)
C6—C7—C18	109.76 (12)	C19—C18—C7	119.69 (13)
C8—C7—C18	111.67 (12)	C23—C18—C7	121.86 (13)
C6—C7—H7A	108.3	C18—C19—C20	121.71 (15)
C8—C7—H7A	108.3	C18—C19—H19A	119.1
C18—C7—H7A	108.3	C20—C19—H19A	119.1
C9—C8—C13	126.22 (14)	C21—C20—C19	118.47 (15)
C9—C8—C7	120.53 (13)	C21—C20—H20A	120.8
C13—C8—C7	113.25 (12)	C19—C20—H20A	120.8
C8—C9—N1	119.20 (14)	C22—C21—C20	121.41 (15)
C8—C9—C12	128.48 (14)	C22—C21—O4	116.50 (14)
N1—C9—C12	112.32 (13)	C20—C21—O4	122.09 (15)
C4—C10—H10A	109.5	C21—C22—C23	118.99 (15)
C4—C10—H10B	109.5	C21—C22—H22A	120.5
H10A—C10—H10B	109.5	C23—C22—H22A	120.5
C4—C10—H10C	109.5	C22—C23—C18	120.93 (15)
H10A—C10—H10C	109.5	C22—C23—H23A	119.5
H10B—C10—H10C	109.5	C18—C23—H23A	119.5
C4—C11—H11A	109.5	F1—C24—F2	106.55 (17)
C4—C11—H11B	109.5	F1—C24—O4	107.35 (17)
H11A—C11—H11B	109.5	F2—C24—O4	110.76 (17)
C4—C11—H11C	109.5	F1—C24—H24A	110.7
H11A—C11—H11C	109.5	F2—C24—H24A	110.7
H11B—C11—H11C	109.5	O4—C24—H24A	110.7
C9—C12—H12A	109.5		
			
C9—N1—C1—C6	13.6 (2)	C13—C8—C9—C12	−7.0 (3)
C9—N1—C1—C2	−168.22 (14)	C7—C8—C9—C12	173.21 (15)
C6—C1—C2—C3	−25.9 (2)	C1—N1—C9—C8	−13.9 (2)
N1—C1—C2—C3	155.98 (14)	C1—N1—C9—C12	165.30 (14)
C1—C2—C3—C4	50.56 (18)	C14—O3—C13—O2	−1.8 (2)
C2—C3—C4—C5	−53.48 (18)	C14—O3—C13—C8	178.30 (14)
C2—C3—C4—C10	−173.70 (14)	C9—C8—C13—O2	−178.39 (17)
C2—C3—C4—C11	64.52 (17)	C7—C8—C13—O2	1.4 (2)
C10—C4—C5—O1	−31.4 (2)	C9—C8—C13—O3	1.5 (2)
C3—C4—C5—O1	−151.72 (15)	C7—C8—C13—O3	−178.68 (13)
C11—C4—C5—O1	87.49 (18)	C13—O3—C14—C15	−174.28 (15)
C10—C4—C5—C6	151.64 (15)	O3—C14—C15—C16	−64.8 (2)
C3—C4—C5—C6	31.3 (2)	O3—C14—C15—C17	171.65 (16)
C11—C4—C5—C6	−89.44 (17)	C6—C7—C18—C19	−48.25 (19)
N1—C1—C6—C5	−177.73 (14)	C8—C7—C18—C19	74.40 (18)
C2—C1—C6—C5	4.2 (2)	C6—C7—C18—C23	130.33 (15)
N1—C1—C6—C7	8.5 (2)	C8—C7—C18—C23	−107.02 (16)
C2—C1—C6—C7	−169.55 (14)	C23—C18—C19—C20	−1.0 (2)
O1—C5—C6—C1	176.01 (15)	C7—C18—C19—C20	177.62 (15)
C4—C5—C6—C1	−7.1 (2)	C18—C19—C20—C21	−1.3 (3)
O1—C5—C6—C7	−10.1 (2)	C19—C20—C21—C22	2.8 (3)
C4—C5—C6—C7	166.76 (14)	C19—C20—C21—O4	−176.98 (16)
C1—C6—C7—C8	−26.47 (19)	C24—O4—C21—C22	−142.79 (17)
C5—C6—C7—C8	159.58 (13)	C24—O4—C21—C20	37.0 (2)
C1—C6—C7—C18	96.98 (16)	C20—C21—C22—C23	−2.1 (3)
C5—C6—C7—C18	−76.97 (17)	O4—C21—C22—C23	177.71 (14)
C6—C7—C8—C9	26.2 (2)	C21—C22—C23—C18	−0.2 (2)
C18—C7—C8—C9	−96.17 (17)	C19—C18—C23—C22	1.8 (2)
C6—C7—C8—C13	−153.64 (13)	C7—C18—C23—C22	−176.84 (14)
C18—C7—C8—C13	84.02 (15)	C21—O4—C24—F1	151.23 (16)
C13—C8—C9—N1	172.06 (15)	C21—O4—C24—F2	−92.82 (19)
C7—C8—C9—N1	−7.7 (2)		

### Hydrogen-bond geometry (Å, º)

*Cg*3 is the centroid the benzene ring of the 4-(4-difluoromethoxyphenyl group of the title compound.

**Table:**

*D*—H···*A*	*D*—H	H···*A*	*D*···*A*	*D*—H···*A*
N1—H1*N*···O1^i^	0.87 (2)	1.98 (2)	2.8426 (17)	173 (2)
C12—H12*A*···O3	0.98	2.40	2.817 (2)	105
C19—H19*A*···O2^i^	0.95	2.43	3.100 (2)	127
C3—H3*A*···*Cg*3^ii^	0.99	2.84	3.7345 (18)	151

Symmetry codes: (i) *x*−1/2, −*y*+1, *z*; (ii) −*x*+1, −*y*+1, *z*+1/2.

## References

[bb1] Abo Al-Hamd, M. G., Tawfik, H. O., Abdullah, O., Yamaguchi, K., Sugiura, M., Mehany, A. B. M., El-Hamamsy, M. H. & El-Moselhy, T. F. (2023). *J. Enzyme Inhib. Med. Chem.* **38**, 2241674. https://doi. org/10.1080/14756366.2023.2241674. 10.1080/14756366.2023.2241674 PMC10408569 37548154

[bb2] Batista, V. F., Pinto, D. C. G. A. & Silva, A. M. S. (2016). *ACS Sustainable Chem. Eng.* **4**, 4064–4078.

[bb3] Bruker (2018). *APEX2* and *SAINT*. Bruker AXS Inc., Madison, Wisconsin, USA.

[bb4] Cremer, D. & Pople, J. A. (1975). *J. Am. Chem. Soc.* **97**, 1354–1358.

[bb5] Farrugia, L. J. (2012). *J. Appl. Cryst.* **45**, 849–854.

[bb6] Groom, C. R., Bruno, I. J., Lightfoot, M. P. & Ward, S. C. (2016). *Acta Cryst.* B**72**, 171–179. 10.1107/S2052520616003954 PMC4822653 27048719

[bb7] Gündüz, M. G., Butcher, R. J., Öztürk Yildirim, S., El-Khouly, A., Şafak, C. & Şimşek, R. (2012). *Acta Cryst.* E**68**, o3404–o3405. 10.1107/S1600536812046909 PMC3588994 23476230

[bb8] Krause, L., Herbst-Irmer, R., Sheldrick, G. M. & Stalke, D. (2015). *J. Appl. Cryst.* **48**, 3–10. 10.1107/S1600576714022985 PMC4453166 26089746

[bb9] Längle, D., Werner, T. R., Wesseler, F., Reckzeh, E., Schaumann, N., Drowley, L., Polla, M., Plowright, A. T., Hirt, M. N., Eschenhagen, T. & Schade, D. (2019). *ChemMedChem*, **14**, 810–822. 10.1002/cmdc.201900036 30768867

[bb10] Linden, A., Gündüz, M. G., Şimşek, R. & Şafak, C. (2006). *Acta Cryst.* C**62**, o227–o230. 10.1107/S0108270106007530 16598150

[bb11] Linden, A., Şafak, C., Şimşek, R. & Gündüz, M. G. (2011). *Acta Cryst.* C**67**, o80–o84. 10.1107/S0108270111003362 21285508

[bb12] Linden, A., Şimşek, R., Gündüz, M. & Şafak, C. (2005). *Acta Cryst.* C**61**, o731–o734. 10.1107/S0108270105037066 16330861

[bb13] Mookiah, P., Rajesh, K., Narasimhamurthy, T., Vijayakumar, V. & Srinivasan, N. (2009). *Acta Cryst.* E**65**, o2664. 10.1107/S1600536809039877 PMC2971334 21578275

[bb14] Öztürk Yildirim, S., Butcher, R. J., Gündüz, M. G., El-Khouly, A., Şimşek, R. & Şafak, C. (2013). *Acta Cryst.* E**69**, o40–o41. 10.1107/S1600536812047976 PMC3588325 23476426

[bb15] Parsons, S., Flack, H. D. & Wagner, T. (2013). *Acta Cryst.* B**69**, 249–259. 10.1107/S2052519213010014 PMC3661305 23719469

[bb16] Pehlivanlar, E., Yıldırım, S. Ö., Şimşek, R., Akkurt, M., Butcher, R. J. & Bhattarai, A. (2023). *Acta Cryst.* E**79**, 664–668. 10.1107/S2056989023005455 PMC10439425 37601569

[bb17] Ranjbar, S., Edraki, N., Firuzi, O., Khoshneviszadeh, M. & Miri, R. (2019). *Mol. Divers.* **23**, 471–508. 10.1007/s11030-018-9886-4 30390186

[bb18] Shahraki, O., Khoshneviszadeh, M., Dehghani, M., Mohabbati, M., Tavakkoli, M., Saso, L., Edraki, N. & Firuzi, O. (2020). *Molecules*, **25**, 1839. https://doi. org/10.3390/molecules25081839. 10.3390/molecules25081839 PMC7221826 32316291

[bb19] Sheldrick, G. M. (2015*a*). *Acta Cryst.* A**71**, 3–8.

[bb20] Sheldrick, G. M. (2015*b*). *Acta Cryst.* C**71**, 3–8.

[bb21] Spackman, P. R., Turner, M. J., McKinnon, J. J., Wolff, S. K., Grimwood, D. J., Jayatilaka, D. & Spackman, M. A. (2021). *J. Appl. Cryst.* **54**, 1006–1011. 10.1107/S1600576721002910 PMC8202033 34188619

[bb22] Spek, A. L. (2020). *Acta Cryst.* E**76**, 1–11. 10.1107/S2056989019016244 PMC6944088 31921444

[bb23] Steiger, S. A., Monacelli, A. J., Li, C., Hunting, J. L. & Natale, N. R. (2014). *Acta Cryst.* C**70**, 790–795. 10.1107/S2053229614015617 PMC4174017 25093361

[bb24] Yıldırım, S. Ö., Akkurt, M., Çetin, G., Şimşek, R., Butcher, R. J. & Bhattarai, A. (2022). *Acta Cryst.* E**78**, 798–803. 10.1107/S2056989022007022 PMC9361379 35974826

[bb25] Yıldırım, S. Ö., Akkurt, M., Çetin, G., Şimşek, R., Butcher, R. J. & Bhattarai, A. (2023). *Acta Cryst.* E**79**, 187–191. 10.1107/S205698902300141X PMC9993909 36909987

